# Supplementary material for: Evolution of lasR mutants in polymorphic Pseudomonas aeruginosa populations facilitates chronic infection of the lung
Source: Nat Commun. 2023 Sep 25;14:5976. doi: 10.1038/s41467-023-41704-w (PMC10519970; doi:10.1038/s41467-023-41704-w)
Supplement: Supplementary file 1 — Supplementary Information [file 41467_2023_41704_MOESM1_ESM.pdf]

Evolution of *lasR* mutants in polymorphic *Pseudomonas aeruginosa* populations facilitates chronic infection of the lung

Kelei Zhao[1,*], Xiting Yang[1], Qianglin Zeng[1], Yige Zhang[2], Heyue Li[3], Chaochao Yan[4], Jing Shirley Li[2], Huan Liu[2], Liangming Du[1], Yi Wu[1], Gui Huang[1], Ting Huang[1], Yamei Zhang[1], Hui Zhou[1], Xinrong Wang[1], Yiwen Chu[1,*] and Xikun Zhou[2,*]

[1] Antibiotics Research and Re-evaluation Key Laboratory of Sichuan Province, School of Pharmacy, Affiliated Hospital of Chengdu University, Chengdu University, Chengdu 610106, China;

[2] State Key Laboratory of Biotherapy and Cancer Center, West China Hospital, West China Medical School, Sichuan University, and Collaborative Innovation Center for Biotherapy, Chengdu 610041, China;

[3] Key Laboratory of Bio-resources and Eco-environment, Ministry of Education, College of Life Sciences, Sichuan University, Chengdu 610064, China.

[4] Ecological Restoration and Biodiversity Conservation Key Laboratory of Sichuan Province, Chengdu Institute of Biology, Chinese Academy of Sciences, Chengdu 610041, China.

* Correspondence:

zhaokelei@cdu.edu.cn (K.Z.), chuyiwen@cdu.edu.cn (Y.C.), xikunzhou@scu.edu.cn (X.Z.).

This file includes:

Supplementary Figures S1 to S19

Supplementary Figures

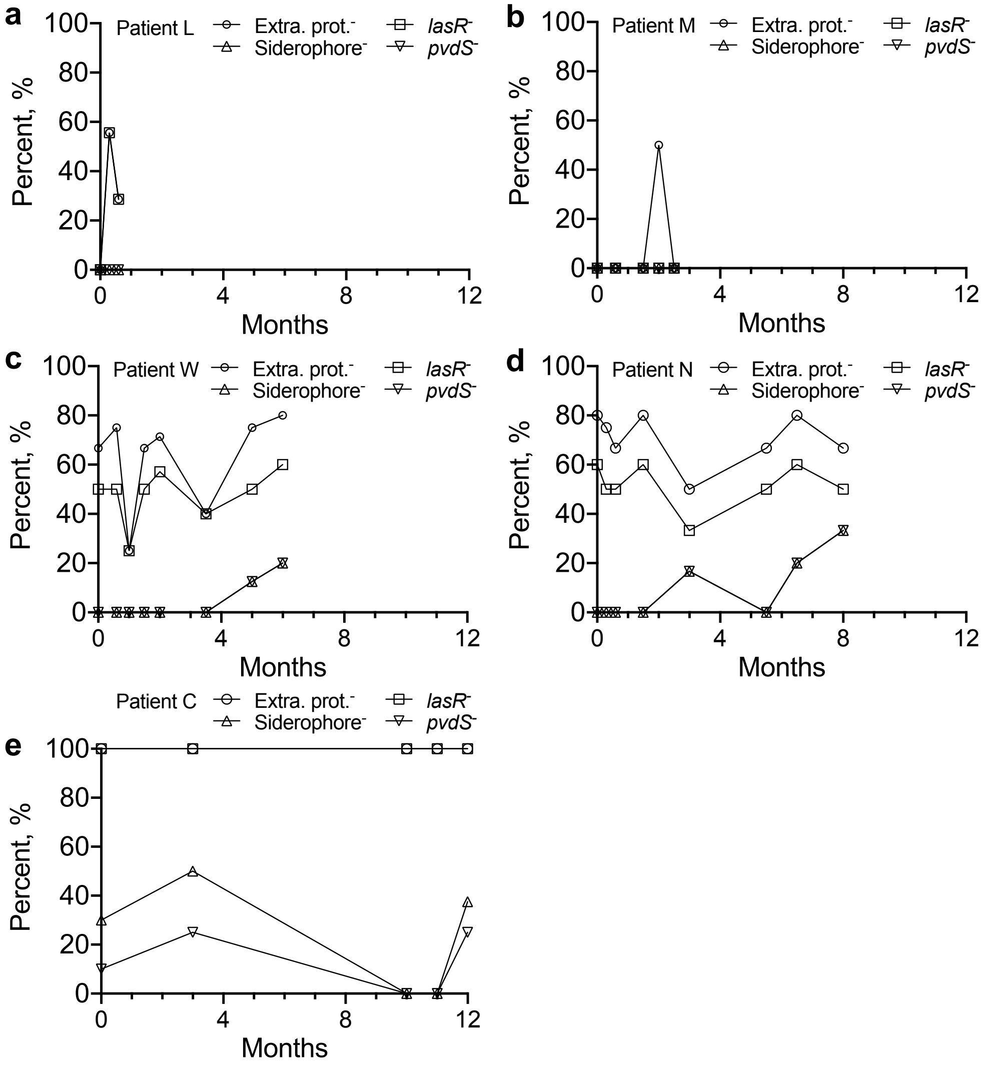

Figure S1. Capacities of *P. aeruginosa* COPD isolates in producing QS-controlled extracellular proteases and siderophores as sampling time increased. The numbers of isolates at different time points are 4, 9 and 7 from the 3 sampling periods of patient L (**a**), 11, 3, 1, 2, and 2 from the 5 sampling periods of patient M (**b**), 6, 4, 4, 6, 7, 5, 8, and 5 from the 8 sampling periods of patient W (**c**), 5, 4, 6, 5, 6, 6, 5, and 6 from the 8 sampling periods of patient N (**d**), and 10, 4, 4, 2, and 8 from the 5 sampling periods of patient C (**e**). Extra. prot.⁻, isolate deficient in producing extracellular protease. *lasR*⁻, *lasR* mutant. siderophore⁻, isolate deficient in producing siderophores. *pvdS*⁻, *pvdS* mutant.

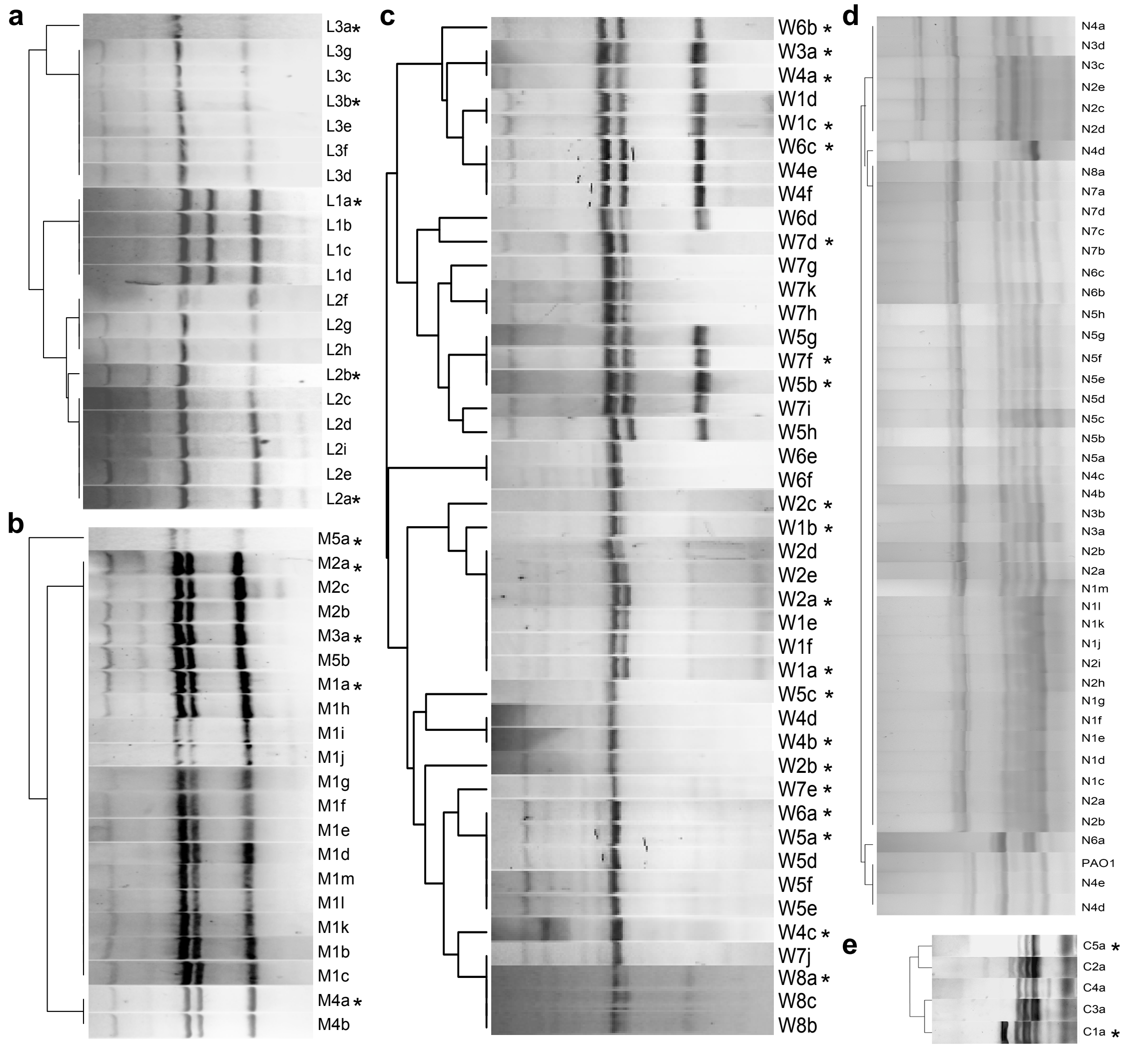

Figure S2. ERIC-PCR-based typing of *P. aeruginosa* COPD isolates from patients (**a**) L, (**b**) M, (**c**) W, (**d**) N, and (**e**) C. Asterisks (*) indicate the isolates selected for whole-genome sequencing.

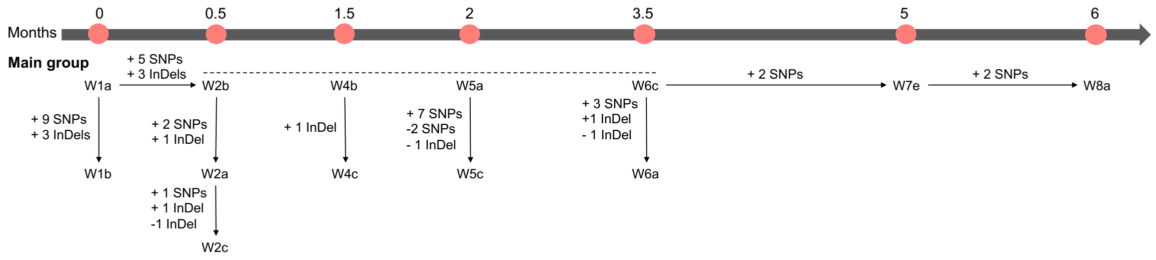

Figure S3. Time-dependent delivery of mutations among the isolates in the main group of *P. aeruginosa* isolates from patient W.

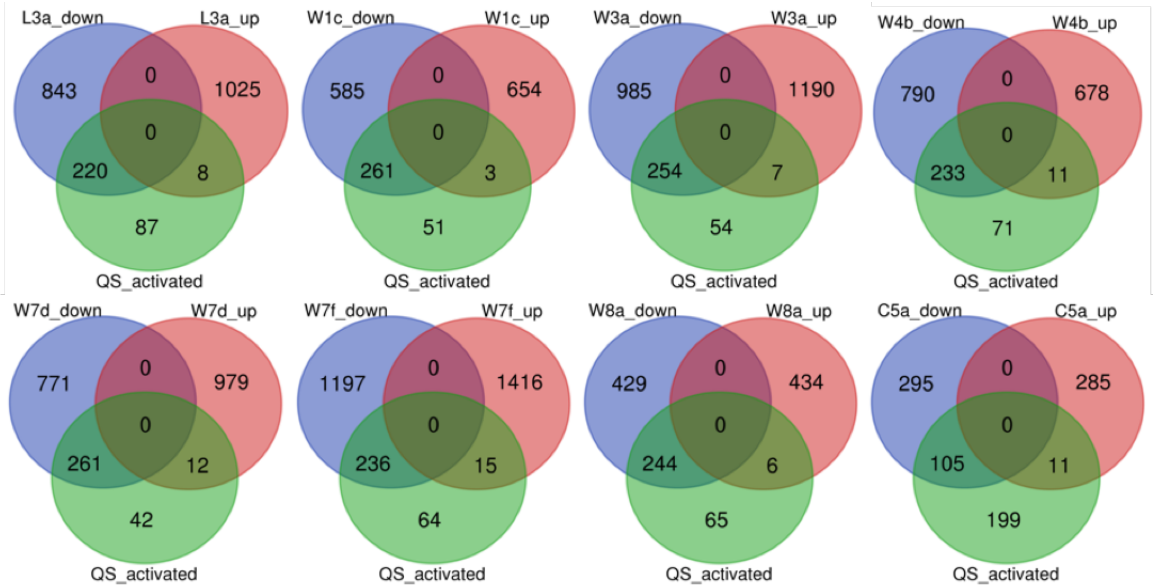

Figure S4. Numbers of genes activated by quorum-sensing (QS) system among the significantly down- and up-regulated (P < 0.05) genes of *P. aeruginosa* COPD isolates compared to their corresponding initial isolates.

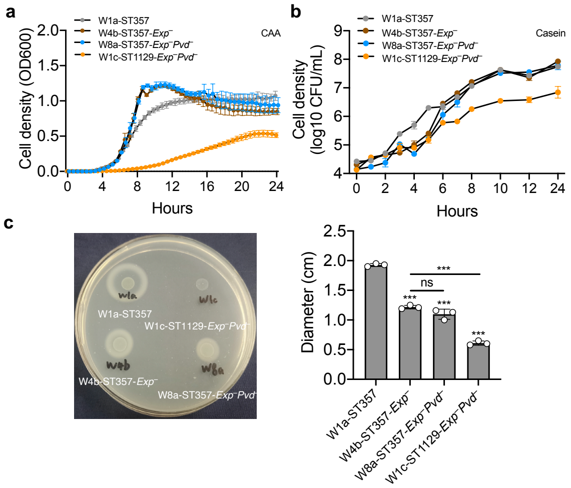

Figure S5. Growth of *P. aeruginosa* isolates from patient W in (**a**) M9-CAA (0.1%) broth, (**b**) M9-casein (0.5%) broth, or on (**c**) M9-casein (0.5%) plate. Data shown are means ± standard deviation (SD) of (**a**) eight, (**b**) four, and (**c**) three independent replicates, respectively. Two tailed unpaired *t*-test. ***, *P* < 0.001. ns, not significant.

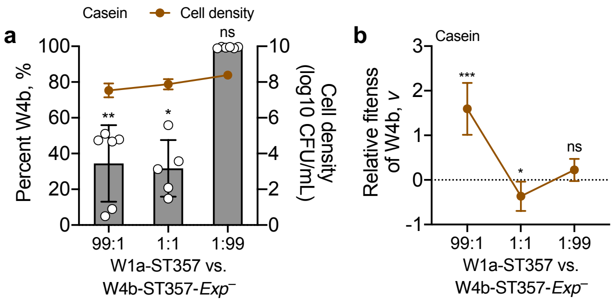

Figure S6. *In vitro* competition of *P. aeruginosa* W1a-ST357 and W4b-ST357-*Exp*⁻ in M9-casein medium. (**a**) Frequency and (**b**) relative fitness of W4b in the coculture for 24 h. Right Y-axis indicates the cell densities of each culture. Data shown are means ± SD of six independent replicates. The value of each column was compared to the initial frequency of corresponding isolate using two tailed unpaired *t*-test. *, $P < 0.05$. **, $P < 0.01$. ***, $P < 0.001$. ns, not significant.

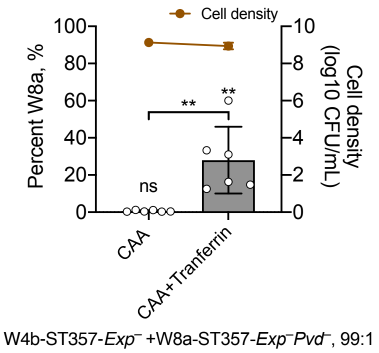

Figure S7. *In vitro* competition of *P. aeruginosa* W4b-ST357-*Exp*$^{-}$ and W8a-ST357-*Exp*$^{-}$*Pvd*$^{-}$ in M9-CAA medium with or without iron limitation. Right Y-axis indicates the cell densities of each culture. Data shown are means ± SD of six independent replicates. The value of each column was compared to the initial frequency of corresponding isolate, or between the two groups using two tailed unpaired *t*-test. **, $P < 0.01$. ns, not significant.

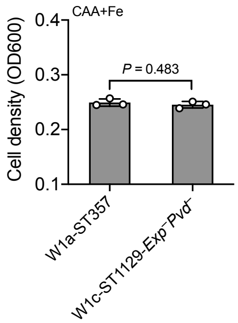

Figure S8. Cell densities of monocultured *P. aeruginosa* W1a-ST357 and W1c-ST1129-*Exp*$^-$*Pvd*$^-$ in M9-CAA medium containing 50 μM of $FeCl_3$ for 24 h. Data shown are means ± SD of three independent replicates. Two tailed unpaired *t*-test.

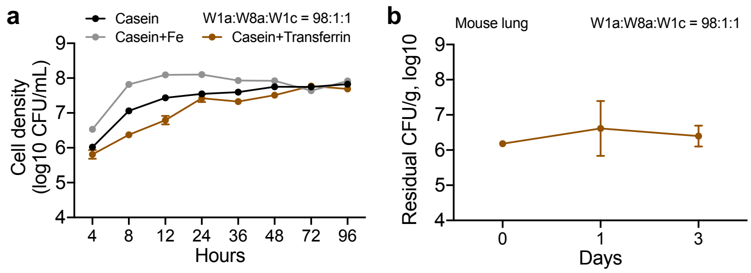

Figure S9. Growth curves of *P. aeruginosa* W1a-ST357, W8a-ST357-*Exp*⁻*Pvd*⁻, and W1c-ST1129-*Exp*⁻*Pvd*⁻ cocultured under different conditions. (**a**) Cell densities of W1a, W1c, and W8a sub-cocultured in different media from an initial ratio of 98:1:1. The culture media were refreshed at 24 h interval and the experiment were stopped when the frequency of each isolate in the culture was relatively stable. Data shown are means ± SD of six independent replicates. (**b**) Residual colony forming units (CFU) in mouse lungs coinfected by W1a-ST357, W8a-ST357-*Exp*⁻*Pvd*⁻, and W1c-ST1129-*Exp*⁻*Pvd*⁻ from an initial ratio of 98:1:1. Data shown are means ± SD of five independent replicates.

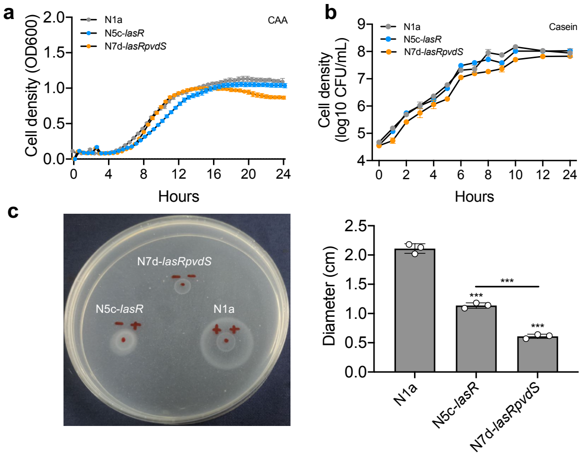

Figure S10. Growth of *P. aeruginosa* isolates from patient N in (**a**) M9-CAA (0.1%) broth, (**b**) M9-casein (0.5%) broth, or on (**c**) M9-casein (0.5%) plate. Data shown are means ± standard deviation (SD) of (**a**) eight, (**b**) three, and (**c**) three independent replicates, respectively. Two tailed unpaired *t*-test. ***, $P < 0.001$.

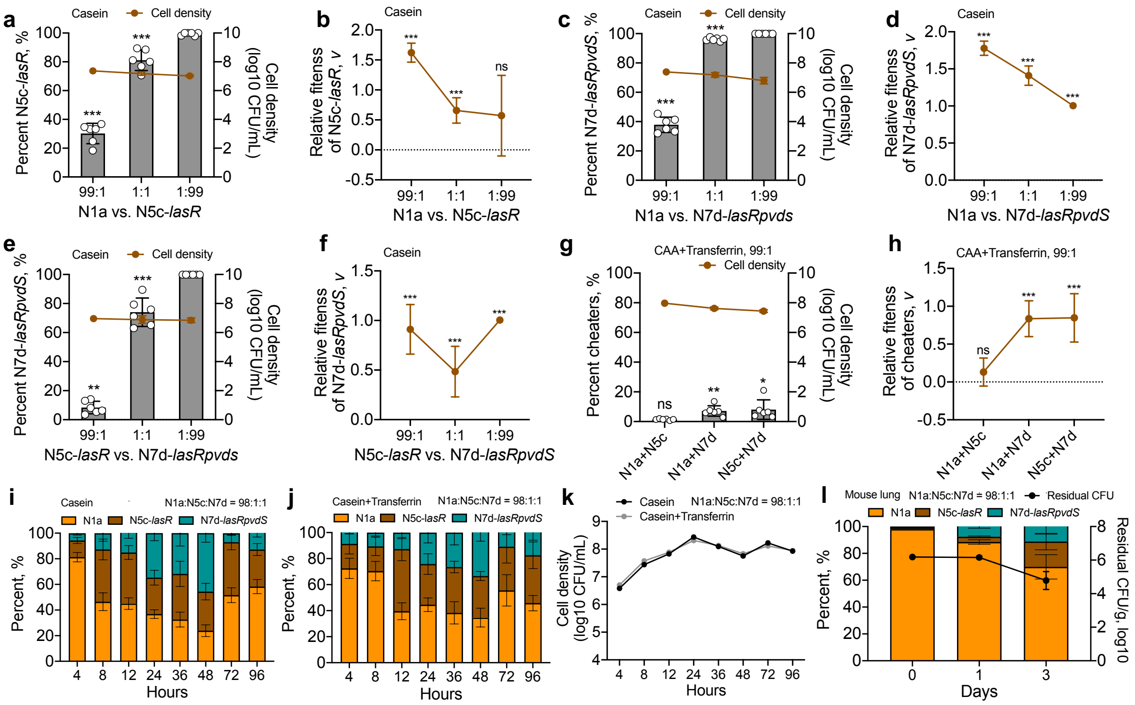

Figure S11. *In vitro* and *in vivo* competitions of *P. aeruginosa* isolates from patient N under different conditions. *P. aeruginosa* isolates N1a (*lasR*-intact), N5c-*lasR* (*lasR* mutant), and N7d-*lasRpvdS* (*lasR-pvdS* mutant) were cocultured in double or triple in 2 mL of M9 minimal growth medium supplemented with different carbon sources and iron levels from different initial ratios. Frequencies and relative fitness of N5c-*lasR* or N7d-*lasRpvdS* in double cocultures of (**a** and **b**) N1a+N5c-*lasR*, (**c** and **d**) N1a+N7d-*lasRpvdS*, or (**e** and **f**) N5c-*lasR*+N7d-*lasRpvdS* in M9-casein medium for 24h. (**g**) Frequencies and (**h**) relative fitness of the isolates with an initial frequency of 1% in double cocultures of N1a+N5c-*lasR*, N1a+N7d-*lasRpvdS*, or N5c-*lasR*+N7d-*lasRpvdS* in iron-limiting M9-CAA medium for 24h. Right Y-axis indicates the cell densities of each culture. The value of each column was compared to the initial frequency of corresponding isolate using two tailed unpaired *t*-test. *, $P < 0.05$. **, $P < 0.01$. ***, $P < 0.001$. Dynamic changes in the frequencies of N1a, N5c-*lasR* and N7d-*lasRpvdS* during the coevolution in (**i**) M9-casein, (**j**) iron-limiting M9-casein, and (**l**) mouse lungs from an initial ratio of 98:1:1. (**k**) Cell densities of N1a, N5c-*lasR* and N7d-*lasRpvdS* during coevolution. The culture media were refreshed at 24 h interval and the experiments were stopped when the frequency of each isolate in the culture was relatively stable. Data shown are means ± standard deviation (SD) of (**a-k**) six and (**l**) three independent replicates.

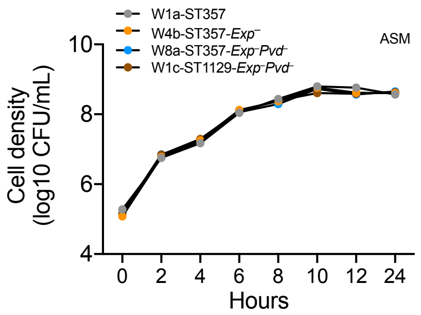

Figure S12. Growth curves of *P. aeruginosa* isolates from patient W in 2 mL of artificial sputum medium (ASM). Data shown are line and means (SD) of three independent replicates.

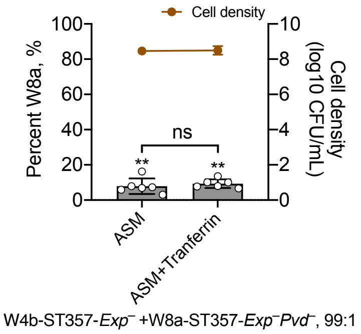

Figure S13. *In vitro* competition of *P. aeruginosa* W4b-ST357-*Exp*⁻ and W8a-ST357-*Exp*⁻*Pvd*⁻ in ASM with or without iron limitation. Right Y-axis indicates the cell densities of each culture. Data shown are means ± SD of six independent replicates. The value of each column was compared to the initial frequency of corresponding isolate, or between the two groups using two tailed unpaired *t*-test. **, $P < 0.01$. ns, not significant.

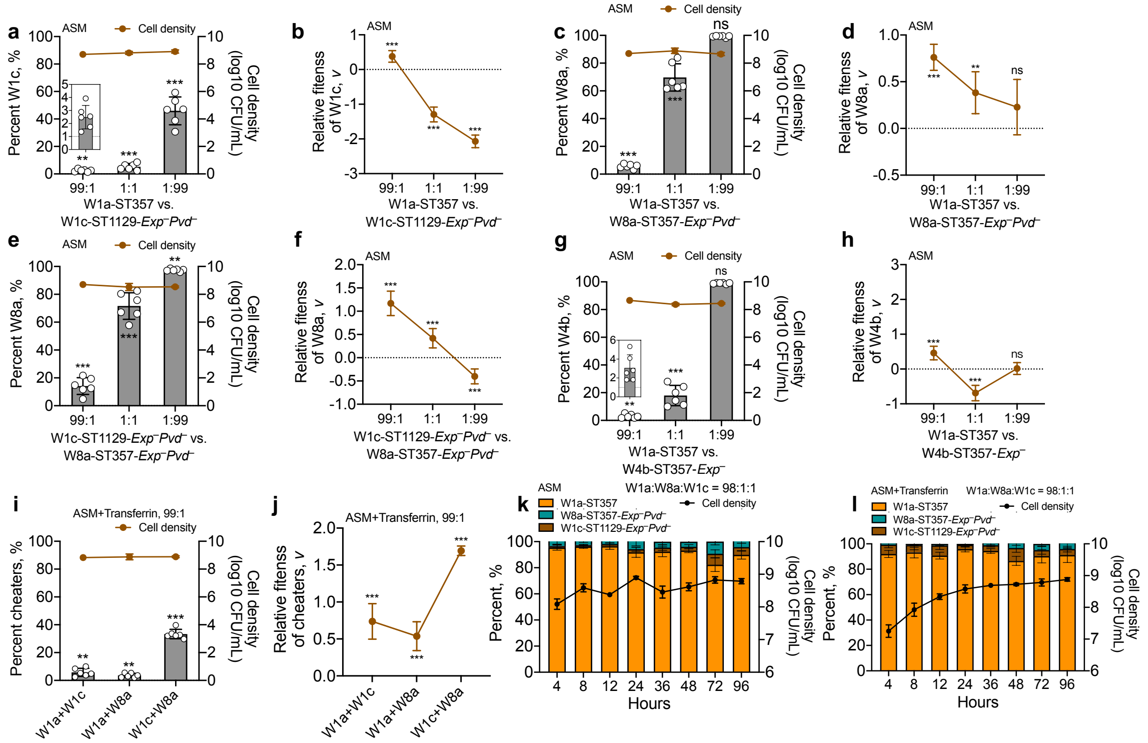

Figure S14. *In vitro* and *in vivo* competitions of *P. aeruginosa* COPD isolates in ASM containing different levels of iron. Frequencies and relative fitness of W1c-ST1129-*Exp*$^-$*Pvd*$^-$ or W8a-ST357-*Exp*$^-$*Pvd*$^-$ in double cocultures of (**a** and **b**) W1a-ST357+W1c-ST1129-*Exp*$^-$*Pvd*$^-$, (**c** and **d**) W1a-ST357+W8a-ST357-*Exp*$^-$*Pvd*$^-$, (**e** and **f**) W1c-ST1129-*Exp*$^-$Pvd$^-$ +W8a-ST357-*Exp*$^-$*Pvd*$^-$, or (**g** and **h**) W1a-ST357+W4b- ST357-*Exp*$^-$ in ASM broth for 24h. (**i**) Frequencies and (**j**) relative fitness of the isolates with an initial frequency of 1% in cocultures of W1a-ST357+W1c-ST1129-*Exp*$^-$*Pvd*$^-$, W1a-ST357+W8a-ST357-*Exp*$^-$*Pvd*$^-$, or W1c-ST1129-*Exp*$^-$*Pvd*$^-$+W8a-ST357-*Exp*$^-$*Pvd*$^-$ in iron-limitation ASM for 24h. Dynamic changes in the frequencies of W1a-ST357, W1c-ST1129-*Exp*$^-$*Pvd*$^-$, and W8a-ST357-*Exp*$^-$*Pvd*$^-$ during the coevolution of the three isolates in (**k**) ASM or (**l**) iron-limiting ASM from an initial ratio of 98:1:1. The culture media were refreshed at 24 h interval and the experiment was stopped when the frequency of each isolate in the culture was relatively stable. Right Y-axis indicates the cell densities of each culture. The value of each column was compared to the initial frequency of corresponding isolate using two tailed unpaired *t*-test. *, $P < 0.05$. **, $P < 0.01$. ***, $P < 0.001$. Data shown are means ± standard deviation (SD) of six independent replicates.

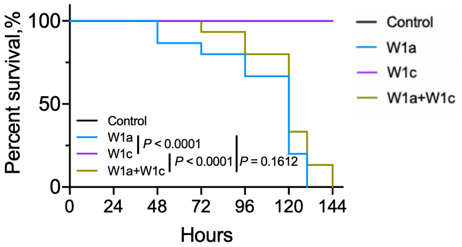

Figure S15. Slow-killing of *Caenorhabditis elegans* infection model (15 nematodes per group) using *P. aeruginosa* isolates W1a-ST357, W1c-ST1129-*Exp*$^{-}$*Pvd*$^{-}$, and 1:1 mixture of them. The survival curves of *C. elegans* and were compared by using Log-rank (Mantel-Cox) test.

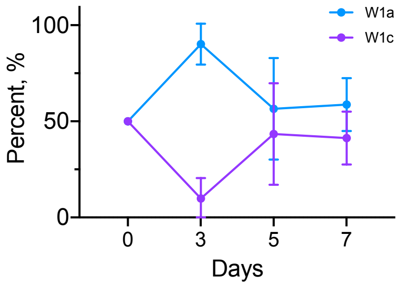

Figure S16. Proportion changes of *P. aeruginosa* isolates W1a-ST357 and W1c-ST1129-*Exp*$^{-}$*Pvd*$^{-}$ in the lungs of mice chronically infected by 1:1 mixture of W1a-ST357 and W1c-ST1129-*Exp*$^{-}$*Pvd*$^{-}$. Data shown are means ± SD of three independent replicates.

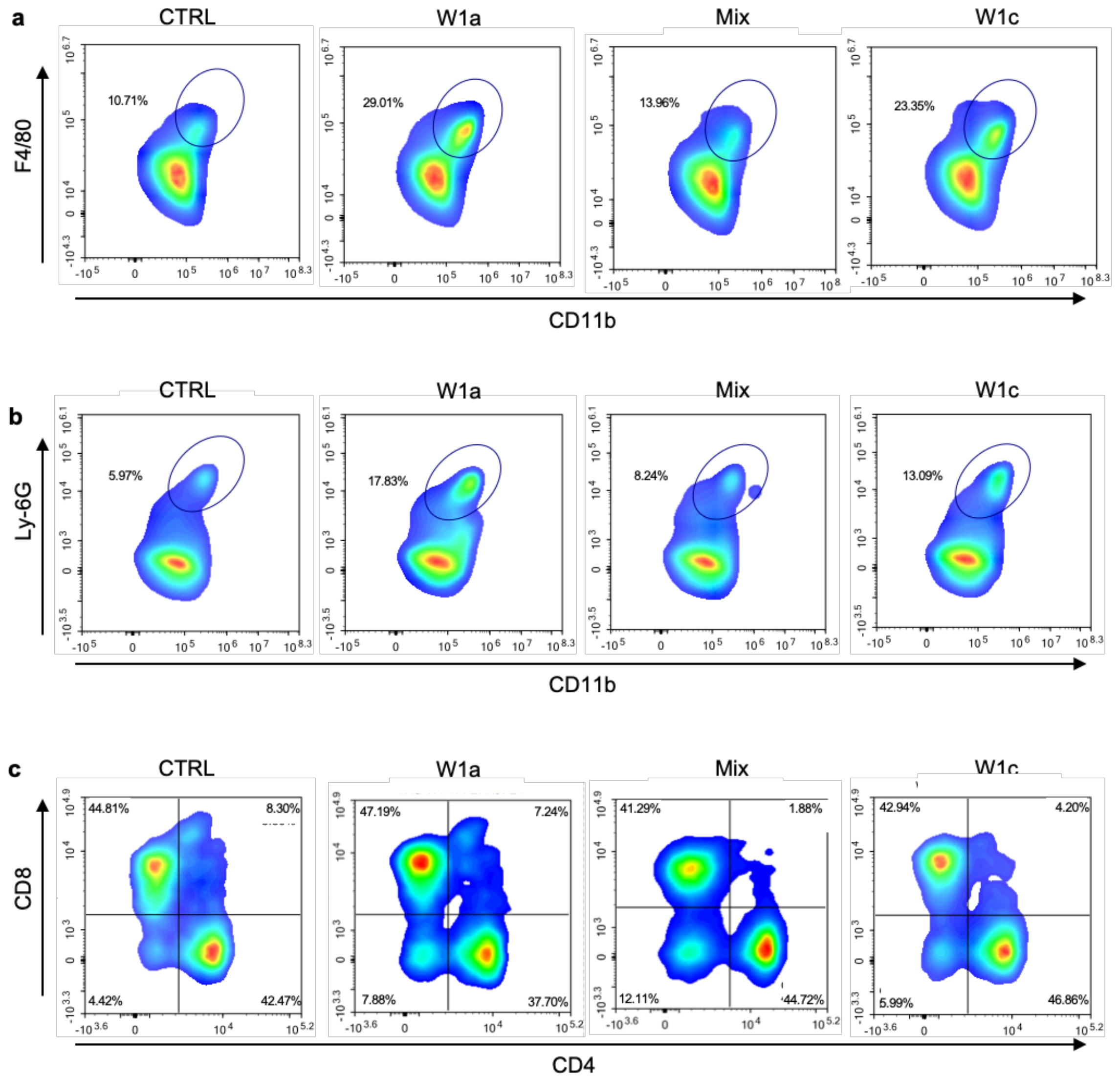

Figure S17. Representative flow cytometry plots of immune cells at day 3. *P. aeruginosa* COPD isolates-infected lung tissues were collected and the proportional changes in inflammatory (**a**) macrophages, (**b**) neutrophils, (**c**) $CD8^+$ T cells and $CD4^+$ T cells were detected under the gate of $CD45^+$ in the different groups. CTRL indicates the control group of mice without *P. aeruginosa* challenge.

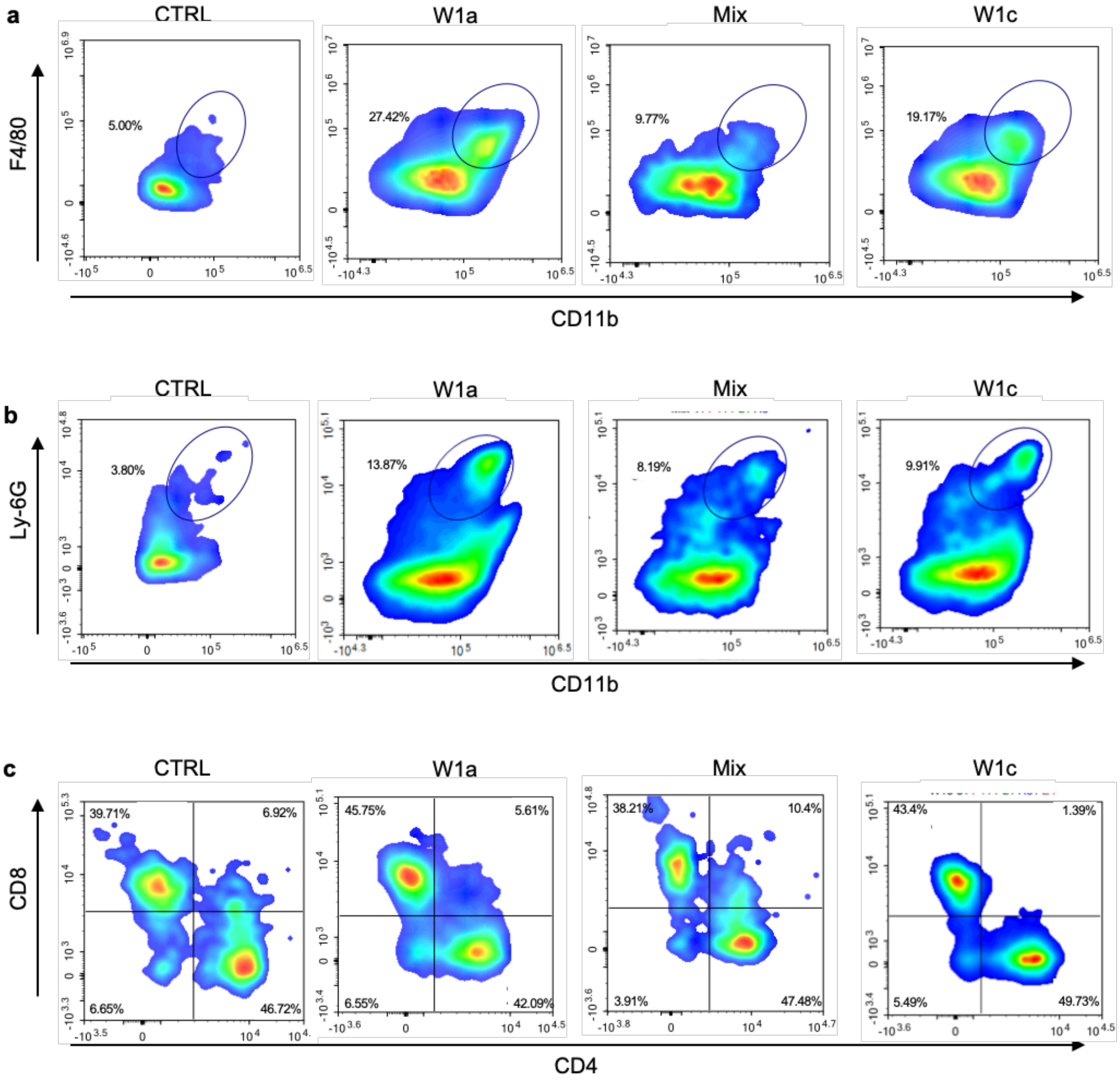

Figure S18. Representative flow cytometry plots of immune cells at day 7. *P. aeruginosa* COPD isolates-infected lung tissues were collected and the proportional changes in inflammatory (**a**) macrophages, (**b**) neutrophils, (**c**) $CD8^+$ T cells and $CD4^+$ T cells were detected under the gate of $CD45^+$ in the different groups. CTRL indicates the control group of mice without *P. aeruginosa* challenge.

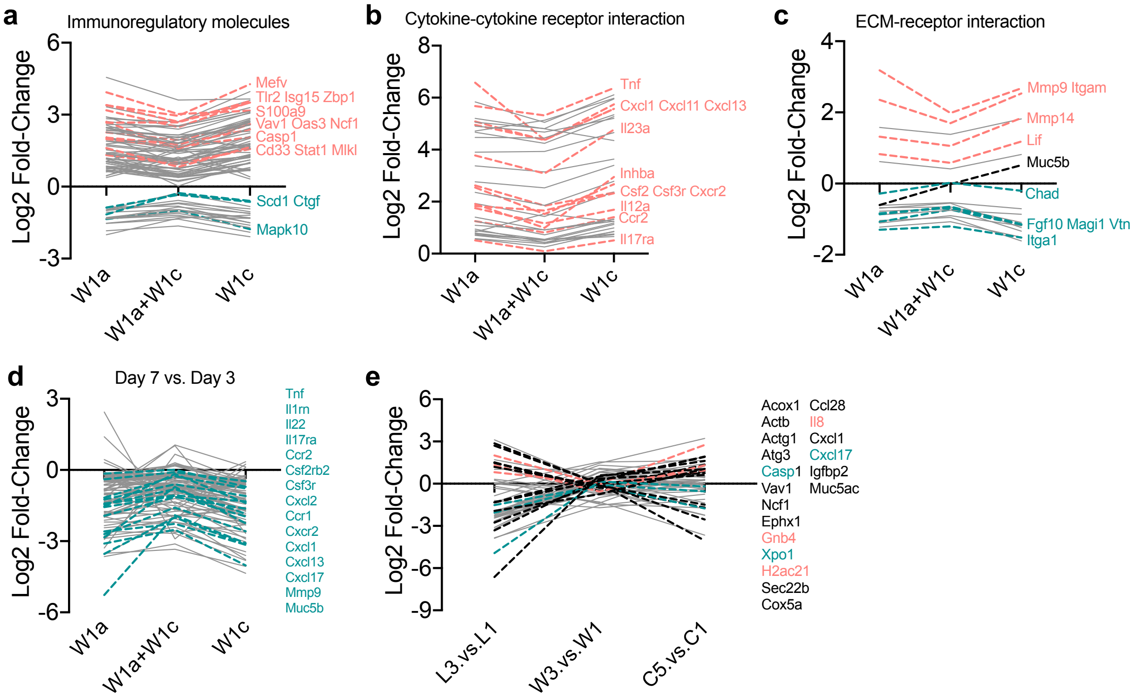

Figure S19. The effects of the interaction of QS-intact and QS-deficient *P. aeruginosa* on differentially expressed genes (different data presentation, related to Figure 5). (**a**-**c**) The fold changes of the differentially expressed genes from *P. aeruginosa* W1a-ST357 and W1c-ST1129-*Exp*$^{-}$*Pvd*$^{-}$ chronically infected mouse lung tissues at day 3. The immune-related genes were sub-clustered into (**a**) immunomodulatory molecules, (**b**) cytokine-receptor interaction and (**c**) ECM-receptor interaction. (**d**) The changes fold of the differentially expressed genes between day 3 and day 7. (**e**) The fold changes of the immune-related differentially expressed proteins between different sampling periods of patient L (the first and the last rounds), patient C (the first and the last rounds), and patient W (rounds 1, 3, 7 and 8).
