# Supplementary material for: Evolution of lasR mutants in polymorphic Pseudomonas aeruginosa populations facilitates chronic infection of the lung
Source: Nat Commun. 2023 Sep 25;14:5976. doi: 10.1038/s41467-023-41704-w (PMC10519970; doi:10.1038/s41467-023-41704-w)
Supplement: Supplementary file 3 — Description of Additional Supplementary Files [file 41467_2023_41704_MOESM3_ESM.pdf]

Description of Additional Supplementary Files:

Supplementary Dataset 1: Assembly information of P. aeruginosa isolates from four COPD patients by using PAO1 genome as the reference.

Supplementary Dataset 2: KEGG terms enriched by significantly down- and up-regulated genes in evolved P. aeruginosa COPD isolates compared to their corresponding initial isolates ($p < 0.05$).

Supplementary Dataset 3: Expression of QS-activated and virulence-related genes in evolved P. aeruginosa COPD isolates compared to their corresponding initial isolates. Data shown are log2 fold change, $p < 0.05$.
