# Supplementary material for: Evolution of lasR mutants in polymorphic Pseudomonas aeruginosa populations facilitates chronic infection of the lung
Source: Nat Commun. 2023 Sep 25;14:5976. doi: 10.1038/s41467-023-41704-w (PMC10519970; doi:10.1038/s41467-023-41704-w)
Supplement: Supplementary file 7 — Reporting summary [file 41467_2023_41704_MOESM7_ESM.pdf]

nature portfolio

Corresponding author(s): Xikun Zhou

Last updated by author(s): Aug 26, 2023

Reporting Summary

Nature Portfolio wishes to improve the reproducibility of the work that we publish. This form provides structure for consistency and transparency in reporting. For further information on Nature Portfolio policies, see our Editorial Policies and the Editorial Policy Checklist.

Statistics

For all statistical analyses, confirm that the following items are present in the figure legend, table legend, main text, or Methods section.

n/a	Confirmed	
☐	☒	The exact sample size (n) for each experimental group/condition, given as a discrete number and unit of measurement
☐	☒	A statement on whether measurements were taken from distinct samples or whether the same sample was measured repeatedly
☐	☒	The statistical test(s) used AND whether they are one- or two-sided<br>*Only common tests should be described solely by name; describe more complex techniques in the Methods section.*
☐	☒	A description of all covariates tested
☐	☒	A description of any assumptions or corrections, such as tests of normality and adjustment for multiple comparisons
☐	☒	A full description of the statistical parameters including central tendency (e.g. means) or other basic estimates (e.g. regression coefficient) AND variation (e.g. standard deviation) or associated estimates of uncertainty (e.g. confidence intervals)
☐	☒	For null hypothesis testing, the test statistic (e.g. F, t, r) with confidence intervals, effect sizes, degrees of freedom and P value noted<br>*Give P values as exact values whenever suitable.*
☒	☐	For Bayesian analysis, information on the choice of priors and Markov chain Monte Carlo settings
☒	☐	For hierarchical and complex designs, identification of the appropriate level for tests and full reporting of outcomes
☒	☐	Estimates of effect sizes (e.g. Cohen's d, Pearson's r), indicating how they were calculated

Our web collection on statistics for biologists contains articles on many of the points above.

Software and code

Policy information about availability of computer code

Data collection	Flow cytometry: BD FACSymphony analyze (BD Biosciences); Growth curve: SPECTROstar Nano® (BMG Labtech)
Data analysis	Flow cytometric analysis: FlowJo software (v10.4 for Mac OS X); ImageJ software (v1.8.0); Kaplan–Meier survival analysis and statistical analyses: GraphPad Prism (v9.0) ; Bacterial growth data analyses: Microsoft excel (v2019 for Mac OS X); GraphPad Prism (v9.0); Comparative genomic analyses: SOAP denovo v2.04, SPAdes, ABySS, Gapclose v1.12, GeneMarkS, MUMmer, LASTZ, SnpEff v4.3, kSNP v3.0; Transcriptomic analyses:Bowtie 2 v2.2.3, HTSeq v0.9.1, DESeq 2, KOBAS v2.0, GOseq R, Hisat2 v2.0.5, featureCounts v1.5.0-p3, and clusterProfiler R package

For manuscripts utilizing custom algorithms or software that are central to the research but not yet described in published literature, software must be made available to editors and reviewers. We strongly encourage code deposition in a community repository (e.g. GitHub). See the Nature Portfolio guidelines for submitting code & software for further information.

Data

Policy information about availability of data

All manuscripts must include a data availability statement. This statement should provide the following information, where applicable:

- Accession codes, unique identifiers, or web links for publicly available datasets
- A description of any restrictions on data availability
- For clinical datasets or third party data, please ensure that the statement adheres to our policy

The high-throughput sequencing data are deposited in the NCBI BioProject database under accession number PRJNA846307. Additional data that support the findings of this study are provided in the supplementary files. Source data are provided with this paper.

Research involving human participants, their data, or biological material

Policy information about studies with human participants or human data. See also policy information about sex, gender (identity/presentation), and sexual orientation and race, ethnicity and racism.

Reporting on sex and gender	The respiratory tract samples were collected from 18 men and 7 women.
Reporting on race, ethnicity, or other socially relevant groupings	Local Chinese patients in the Affiliated Hospital of Chengdu University.
Population characteristics	A total of 25 patients (56 to 92 years old) who were diagnosed as COPD and P. aeruginosa-positive (but negative in the past whole year) hospitalized in the Affiliated Hospital of Chengdu University were enrolled for longitudinal collection of P. aeruginosa.
Recruitment	All samples were collected from the patients who had provided informed consent at the Affiliated Hospital of Chengdu University, and all the related procedures were performed with the approval of the internal review and local ethics committee of the Affiliated Hospital of Chengdu University. Only the patients who were diagnosed as COPD and P. aeruginosa-positive (but negative in the past whole year) were enrolled.
Ethics oversight	BALs and sputum samples were obtained from the COPD patients hospitalized in the affiliated hospital of Chengdu University (Chengdu, China). Written informed consents were received from the patients or their immediate family members. The study was approved by the Ethics Committee of the Affiliated Hospital of Chengdu University (PJ2020-021-03), and all methods were carried out in accordance with the guidelines and regulations of Chengdu University.

Note that full information on the approval of the study protocol must also be provided in the manuscript.

Field-specific reporting

Please select the one below that is the best fit for your research. If you are not sure, read the appropriate sections before making your selection.

[x] Life sciences [ ] Behavioural & social sciences [ ] Ecological, evolutionary & environmental sciences

For a reference copy of the document with all sections, see nature.com/documents/nr-reporting-summary-flat.pdf

Life sciences study design

All studies must disclose on these points even when the disclosure is negative.

Sample size	For in vitro experiments, the sample size was determined based on pilot experiments with at least 3 independent replicates for the routine phenotypic anlyses, and 6 independent replicates for the cell-cell interaction analyses (Zhao et al., BMC Biol. 2019, 17: 20). For clinical sample analysis or animal experiments, samples size was estimated on the knowledge on good sample size to ensure adequate data for reliable. All experiments included at least 3 independent experiments (Zhao et al., Nat. Microbiol. 2019, 4: 459–469). The number of independent experiment was indicated in each figure legend.
Data exclusions	The data of biological replicate that was identified as a failed experiment were excluded.
Replication	For each experiment, sample size and number of independent experiments are indicated in the appropriate figure legend. In vitro studies are represented at least 3 independent reproducible studies. Animals studies represent at least 3 independent mice.
Randomization	All the samples from each experiment and the animals were randomly allocated to each treatment group. Different treatment groups were processed identically, and animals in different treatment groups were exposed to the same environment.
Blinding	Investigators did not blind to group allocation during experiments and data analysis. However, all the experiments and analyses were carried out without any prior biases and in an objective, rigorous scientific way. Conclusions were made based on statistical significance of the data.

Reporting for specific materials, systems and methods

We require information from authors about some types of materials, experimental systems and methods used in many studies. Here, indicate whether each material, system or method listed is relevant to your study. If you are not sure if a list item applies to your research, read the appropriate section before selecting a response.

Materials & experimental systems

n/a	Involved in the study
☒	☐ Antibodies
☒	☐ Eukaryotic cell lines
☒	☐ Palaeontology and archaeology
☐	☒ Animals and other organisms
☒	☐ Clinical data
☒	☐ Dual use research of concern
☒	☐ Plants

Methods

n/a	Involved in the study
☒	☐ ChIP-seq
☐	☒ Flow cytometry
☒	☐ MRI-based neuroimaging

Animals and other research organisms

Policy information about studies involving animals; ARRIVE guidelines recommended for reporting animal research, and Sex and Gender in Research

Laboratory animals	8-10 week-old female C57BL/6 mice were purchased from Beijing HFK Bioscience Co. All mice were routinely housed in a specific pathogen-free facility at the State Key Laboratory of Biotherapy, Sichuan University, according to the Institutional Animal Care and Use Committee (IACUC). Standard conditions of dark/light cycle (12/12 with lights on at 7 AM and off at 7 PM), ambient temperature (70-71oF) and humidity (50-55%) were used.
Wild animals	No wild animals were used.
Reporting on sex	Female mice were only used in this study
Field-collected samples	No field-collected samples were used in this study.
Ethics oversight	Animal experiments were approved by the Ethics Committee of the State Key Laboratory of Biotherapy (2021559A) and carried out in compliance with institutional guidelines concerning animal use and care of Sichuan University.

Note that full information on the approval of the study protocol must also be provided in the manuscript.

Flow Cytometry

Plots

Confirm that:

- ☒ The axis labels state the marker and fluorochrome used (e.g. CD4-FITC).
- ☒ The axis scales are clearly visible. Include numbers along axes only for bottom left plot of group (a 'group' is an analysis of identical markers).
- ☒ All plots are contour plots with outliers or pseudocolor plots.
- ☒ A numerical value for number of cells or percentage (with statistics) is provided.

Methodology

Sample preparation	Single-cell suspensions were obtained from lung tissues of mice. Briefly, the lungs of mice were placed in a gentle MACS™ C tube (Miltenyi Biotec) and mechanically homogenized in 4 ml RPMI-1640 and 10% FBS containing 0.2 mg/ml collagenase type I/IV using a gentle MACS™ Octo Dissociator with Heaters following the manufacturer's protocol (Miltenyi Biotec). The red blood cells in digested lung tissues were removed using 2 ml red blood cell lysis buffer. Finally, the cell suspensions were incubated with antibodies.
Instrument	BD FACSymphony analyze (BD Biosciences)
Software	FlowJo software (v.10.4 for Mac OS X); ImageJ software (v1.8.0); GraphPad Prism (v9.0) ; Microsoft excel (v2019 for Mac OS X).
Cell population abundance	No FACS-sorted samples were used in this study.
Gating strategy	Starting cells were gated by FSC/SSC gates. Gates indicating boundaries between "positive" and "negative" are according to the isotype staining. Expression of indicated proteins were checked on these populations as indicated in the figures and

figure legends

☐ Tick this box to confirm that a figure exemplifying the gating strategy is provided in the Supplementary Information.
